# Bioherder Generated by *Rhodococcus erythropolis* as a Marine Oil Spill Treating Agent

**DOI:** 10.3389/fmicb.2022.860458

**Published:** 2022-04-29

**Authors:** Miao Yu, Zhiwen Zhu, Bing Chen, Yiqi Cao, Baiyu Zhang

**Affiliations:** Northern Region Persistent Organic Pollutant Control (NRPOP) Laboratory, Faculty of Engineering and Applied Science, Memorial University, St. John's, NL, Canada

**Keywords:** biosurfactant, bioherder, *in situ* burning, *Rhodococcus erythropolis*, marine oil spill response, low temperature, herding effectiveness

## Abstract

There is an urgent call for contingency planning with effective and eco-friendly oil spill cleanup responses. *In situ* burning, if properly applied, could greatly mitigate oil in water and minimize the adverse environmental impacts of the spilled oil. Chemical herders have been commonly used along with *in situ* burning to increase the thickness of spilled oil at sea and facilitate combustion. These chemical surfactant-based agents can be applied to the edges of the oil slick and increase its thickness by reducing the water–oil interfacial tension. Biosurfactants have recently been developed as the next generation of herds with a smaller environmental footprint. In this study, the biosurfactant produced by *Rhodococcus erythropolis* M25 was evaluated and demonstrated as an effective herding agent. The impact of environmental and operational factors (e.g., temperature, herder dose, spilled oil amount, water salinity, and operation location) on its performance was investigated. A five-factor fractional design was applied to examine the importance of these factors and their impact on herding effectiveness and efficiency. The results of this study showed that higher temperature and a higher dose of herder could result in an increased oil slick thickness changing rate. Differences in water salinity at the same temperature led to the same trend, that is, the herding process effectively goes up with increasing herder–oil ratio (HOR). Further large-scale testing needs to be conducted for evaluating the applicability of the developed bioherder in the field.

## Introduction

The expansion of offshore oil exploration, production, and transportation activities poses an increasing risk of oil spills to the marine environment (Wang et al., 2022). Therefore, mounting concerns have been expressed about the adverse ecological impact of spill incidents (Zhu et al., 2022). *In situ* burning is considered an effective response option through igniting and burning the oil at the spill location in a controlled manner. When properly operated, it can significantly reduce the volume of spilled oil in a short period, minimizing the environmental impacts of the spilled oil and reducing remediation efforts. Previous studies indicated that *in situ* burning could remove more than 90% of the spilled oil (Fritt-Rasmussen et al., 2012). However, oil floating on the sea surface needs to be 2–3 mm thick to counter heat loss at sea and provide sufficient oil vaporization to maintain oil burning (Al-Majed et al., 2012). Techniques that can be used to increase the thickness of oil slicks are much needed.

Chemical herders, also known as oil-herding surfactants, have been considered as an effective chemical confinement method to contract oil spreading in thicker oil slicks. Amphiphilic oil-herding surfactants, once applied to the oil slick edges, can reduce the interfacial tension between water and air. Unbalanced surface tension between oil–water and dispersant–water resulted in a negative spreading coefficient that could facilitate the herding and thickening of the spread oils with enhanced *in situ* burning efficiency (Ufford et al., 2014). Herders available at the market include hydrocarbon-based herders (e.g., Siltech OP-40, Corexit 9580, original US Navy cold water blend, warm-water herder blend, OC-5), silicone surfactant-based herds (e.g., Siltech OP-40, Silsurf A108, and Silsurf A004D), and second-generation fluorosurfactant PolyFox™ PF151 (Buist et al., 2010; Fingas, 2014). Recent studies have been centered on the effectiveness of oil-herding agents in the arctic environment, where mechanical recovery, such as skimmers, is hard to employ (van Gelderen et al., 2017; Bullock et al., 2019). However, the ecological impact of chemical herders on the marine environment remains to be questioned and hinders their application (Fritt-Rasmussen et al., 2021). Therefore, the development of the next generation of oil-herding agents is flourishing (Huang et al., 2019; Zhou, 2020).

Biosurfactants are surface-active agents produced during microbial growth. As structurally diverse groups of biological molecules, they can be classified into low-molecular-weight biosurfactants (e.g., glycolipids, lipopeptides, and phospholipids) and high-molecular-weight biosurfactants (e.g., polymers). Their amphiphilic structures and biodegradability make them excellent alternatives to chemical surfactants. Trehalolipids are one of the most widely studied glycolipid biosurfactants produced by *Rhodococcus* and other actinomycetes. They can reduce the surface tension of water from 72 to 19–43 mN/m and the water/hexadecane interfacial tension from 43 mN/m to 0.02–15 mN m^−1^ (Kuyukina and Ivshina, 2019). In addition, they have good stability under various environmental conditions, even under high salinities and low temperatures (Table 1) (Zhu et al., 2021). Such properties make their application in the marine environment promising, even in cold-sensitive regions, such as the North Atlantic and Arctic ocean (Cai et al., 2014). The excellent surface activities and environmental compatibility of trehalolipids make them an excellent alternative to chemical surfactants used as oil-herding surfactants. However, the application of trehalolipids as oil-herding biosurfactants or bioherders remains very limited.

**Table 1:** Comparison of the surface activities and toxicities of biosurfactants and chemical surfactants.

**Surfactants**	**Production strain**	**CMC (mg/L)**	**Air–water interfacial tension (mN/m)**	**Oil–water interfacial tension (mN/m)**	**EC50 (mg/L)**	**References**
Trehalose lipids	*Rhodococcus erythropolis* M36	199.16	34.9	N/A	>6150 (15 min) (Microtox^®^)	Cai et al., 2021
Trehalose lipids	*Rhodococcus wratislaviensis* BN38	28.6 (mixture) 24.4 (purified)	32.51 (±0.19)	5.3	N/A	Tuleva et al., 2008
Trehalose lipids	*Rhodococcus ruber* AC 235	N/A	N/A	N/A	650	Effendi et al., 2018
Trehalose lipids	*Rhodococcus erythropolis*	70	29.45	4.45	N/A	Xia et al., 2011
SDS		1731-2308	~35	8.79	18 (EC50-48 h with *Daphnia magna*)	Ahn et al., 2010; Xu et al., 2013; Santos et al., 2018
Triton X-100		106–160	30	2	26 (EC50-48 h with *Daphnia magna*)	Li et al., 2007; Mohammed, 2007; Abu-Ghunmi et al., 2014
Saponin		480	30	1.066	36.5 (EC50-72 h with *Selenastrum capricornutum*)	De Oliveira et al., 2017

The objective of this study is to generate biosurfactant-based bioherder for marine oil spill response. Trehalose lipids generated by *Rhodococcus* were selected as the target biosurfactant for bioherder production. The physiochemical properties of the generated biosurfactant were characterized. At the end, the herding effectiveness was compared with that of the chemical herders to demonstrate its performance.

## Methodologies

### Biosurfactant Production

In that study (Lv et al., 2016), the genetically mutated *Rhodococcus* M25 strain (Supplementary Figure S1) with higher biosurfactant productivity was selected. The strain was cultured on Marine Agar for 3 days and then transferred to seeded culture broth following the method described by Zhu et al. (2019). The seeded culture broth was prepared by autoclaving (121°C for 20 min) 3.74 g of BD Difco^TM^ Marine Broth 2216 in 100 ml of distilled water. A loop of *Rhodococcus* M25 was then collected from the agar plate and transferred into a 250-ml Erlenmeyer flask containing 100 ml of autoclaved seed culture. After 24 h of incubation at 200 rpm under 30°C, the seed culture broth with *Rhodococcus* M25 could be used as an inoculum at the 2% (v/v) level for biosurfactant production.

The biosurfactant production medium was listed as follows: MgSO_4_, 0.2 g; CaCl_2_·2H_2_O, 0.05 g; KH_2_PO_4_, 3.4 g; K_2_HPO_4_·3H_2_O, 4.4 g; (NH_4_)_2_NO_3_, 1 g; FeCl_3_, 0.05 g; Glucose, 1 g; and NaCl, 26 g in 1 L of distilled water, with 3% (v/v) diesel. Five hundred milliliters of culture medium was added to a 1-L Erlenmeyer flask and the *Rhodococcus* M25 inoculum was inoculated into the flask at a ratio of 2% (v/v). The culture broth was incubated in a shaking incubator (200 rpm) at 30°C. After 7 days of incubation, the culture broth was collected and subjected to extraction and purification following the pre-developed protocol (Cai et al., 2016). In general, a thick emulsion layer was formed on the top layer after incubation. To break the emulsion, the culture broth was collected for freeze-and-thaw treatment. The yellowish middle layer with biosurfactants was collected and washed first with petroleum ether to remove oil and then added with sodium sulfate to absorb water. This step was repeated at least three times until no oil and water remained. Then, the biosurfactant was extracted with chloroform and methanol (2:1, v/v). Sonication was performed to detach the cells from the biosurfactants. This procedure was repeated several times until a clear solution appeared in the lower phase. All lower phases were collected, combined, and subjected to concentration by rotary evaporation. The crude biosurfactant products were collected and stored at −20°C for further analysis and testing.

### Characterization of the Generated Biosurfactant

The surface tension of the generated biosurfactant was determined using Goniometers (KRÜSS Scientific). The structure of the generated biosurfactants was characterized using Fourier transform infrared-attenuated total reflection (FTIR-ATR) spectroscopy in the range of 400–4,000 cm^−1^. A data correction of the background spectrum was conducted.

The surface activity of the generated biosurfactant was determined by surface tension, interfacial tension, and contact angle at a biosurfactant concentration of 1 critical micelle concentration (CMC) (0.3 g/L). The surface tension and interfacial tension of generated biosurfactant were measured by the pendant drop method using the DSA-25S goniometer (KRÜSS Scientific). The oil–air contact angle (θ_oa_) with and without the addition of biosurfactant was measured based on the method described by Grate et al. (2012). A 2 cm × 6 cm rectangular silicon plate was cut to fit the contact angle instrument. This plate was immersed in a wash solution prepared at 70°C for 10 min for deep cleaning. The wash solution consisted of 27% ammonium hydroxide (2 ml), 30% hydrogen peroxide (2 ml), and deionized water (10 ml). The plate was then coated with the prepared biosurfactant solution and dried under a stream of nitrogen gas. The contact angle of oil–air measurements was performed with a DSA-25S goniometer (KRÜSS Scientific) using the static sessile drop method. The Arctic North Slope (ANS) was selected as the standard oil for the oil–water interfacial and contact angle measurement.

### Herding Experiment

The herding experiment was conducted following the method developed by Buist et al. (2008) and Gupta et al. (2015). In general, a customized plastic tray was filled with water up to 1.5–2 cm, and crude oil was poured to the center of the water surface. The oil was allowed to spread across the water surface to equilibrium for 20 min, forming a thin film of oil on water. An overhead digital camera was mounted on a customized rig to take photos for further analysis. Crude biosurfactant was used as a bioherder and was added to the system gently from the corner at a herder–oil ratio (HOR) of 1:24, 1:48, 1:72, and 1:144 (v/v). Photographs were taken every minute for at least 20 min.

The herding performance of the generated biosurfactant was evaluated in freshwater and saline water (35 g/L) at different room temperatures. The captured images were processed using the ImageJ software to determine the slick area, which was used to calculate the change rate in thickness with time.


(1) $$\begin{array}{r} \text{Changerate}=\left(\text{Initial area}-\text{Final area}\right)/\text{Initial area}*100\%. \end{array}$$

The camera was set to manual focus mode with a fixed lens. Also, all pictures were taken with the same settings and scales to ensure the objectives had the same sizes. Herding effectiveness is the area change rate between time 0 and 20 min. Bioherder effectiveness was calculated using Equation (1) and was further compared with hydrocarbon—(span) and silicon-based chemical herder (silsurf). The herding efficiency is the area change rate in 1 min after introducing a bioherder into the system.

### Design of Experiments Analysis

Design of experiments (DOE) is a statistical approach to plan, conduct, analyze, and interpret controlled tests, and to evaluate the factors that control the value of a parameter or a group of parameters. Factorial factor design is used to evaluate the impacts and interactions of the variables on the response.

The response of this design is to evaluate herding effectiveness of a bioherder, calculated as the change rate of oil slick thickness over experimental time. The input variables are the factors that impact herding performance, including water temperature (A), water salinity (B), oil (C), herder dose (D), and approach (E). The temperature range (A) was selected between 4°C and 24°C, which represents the water environment in cold and temperate areas. For factor B (salinity), a lower value of 0% represents freshwater, while a higher value of 3.5% represents the marine environment. Saline water was prepared by dissolving 35 g of sea salt (Sigma-Aldrich) in 1 L of distilled water. ANS crude oil was selected as the testing oil. The amount of oil (C) was scaled down from a small-scale bench test (Buist et al., 2010). Different initial oil slick thicknesses were obtained by adding different amounts of ANS crude oil. However, the minimal amount of oil poured onto the water surface was 200 μl. Since the image process software cannot calculate an oil slick less than this thickness, numeric factors were set at the middle level by experimenting with the center points. The herder dose (D) (5, 10, and 15 μl) was set according to the previous study. The impact of the application approach (location of herder applied) was also evaluated. Adding point “1” was located at the lower right corner of a pert dish, while “2” included the current corner “1” and its diagonal corner. Adding a herder at 2 locations required the coordination of hands and eyes. The selected factors are summarized in Table 2.

**Table 2:** Summary of factors.

**Factor**	**Name**	**Units**	**Low actual**	**High actual**	**Low coded**	**High coded**
A	Temperature	°C	4	24	−1	1
B	Salinity	%	0	3.5	−1	1
C	Oil amount	μl	360	720	−1	1
D	Herder dose	μl	5	15	−1	1
E	Approach		1	2	−1	1

## Results and Discussion

### Bioherder Production and Characterization

**Figure 1:**
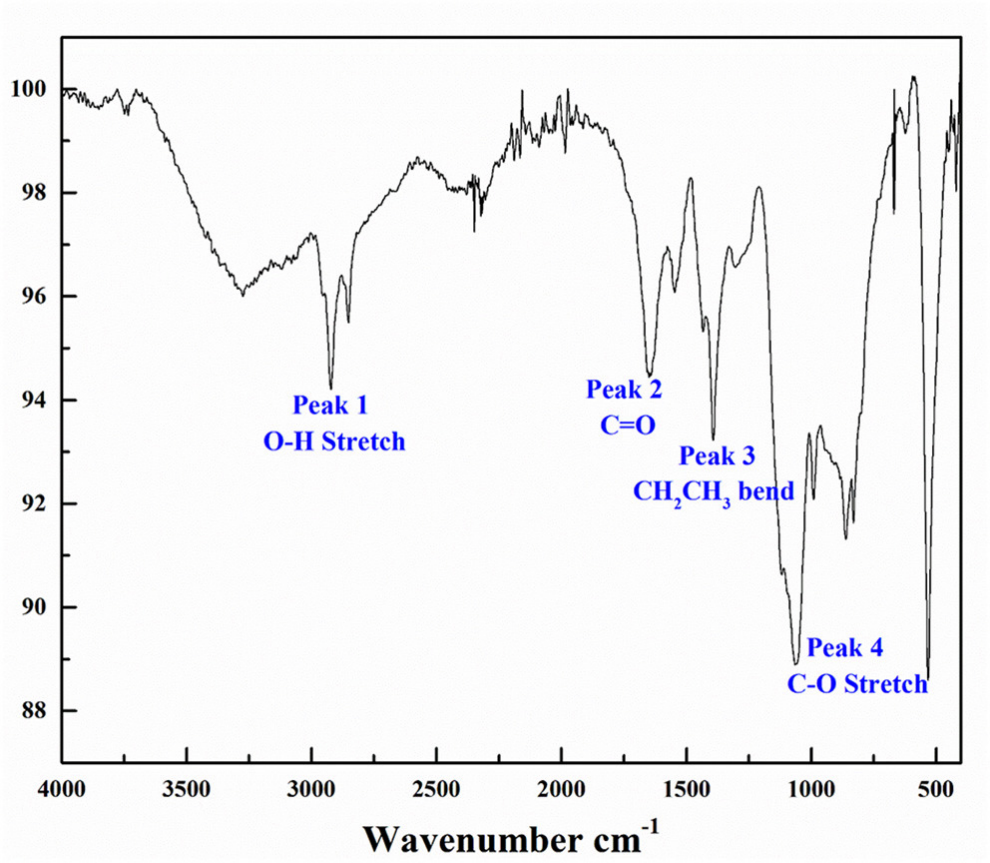
Fourier transform infrared (FTIR) characterization of biosurfactants generated by *Rhodococcus erythropolis* M25.

Bioherders were harvested from production medium (PM) media after 7 days of cultivation, extracted by organic solvents and concentrated by rotary evaporation. The bioherder produced by *Rhodococcus erythropolis* M25 was identified as trehalose lipids (Cai et al., 2016). Most trehalose lipids reported to date are disaccharides, with varying fatty acid chains from C20 to C90 (Franzetti et al., 2010). The α,α-1,1-glycosidic linkage connected the two glucose units. FTIR analysis (Figure 1) of this crude bioherder was conducted to examine the functional groups present in the molecular structure. The FTIR spectrum shows the characteristic vibrational modes of different functional groups, including carboxyl O–H stretch overlapping the C–H stretch at peak 1 (2,500–3,300 cm^−1^), carboxyl –C=O stretch at peak 2 (1,710 cm^−1^), carboxyl C-O stretching at peak 4 (1,085–1,050 cm^−^1), and alkyl groups at peaks 1 and 3 (2,922.37–2,852.11 cm^−1^, alkyl C–H stretch and 1,432.83–1,302.61 cm^−1^, alkyl –C–H bending) (peak 2).

**Figure 2:**
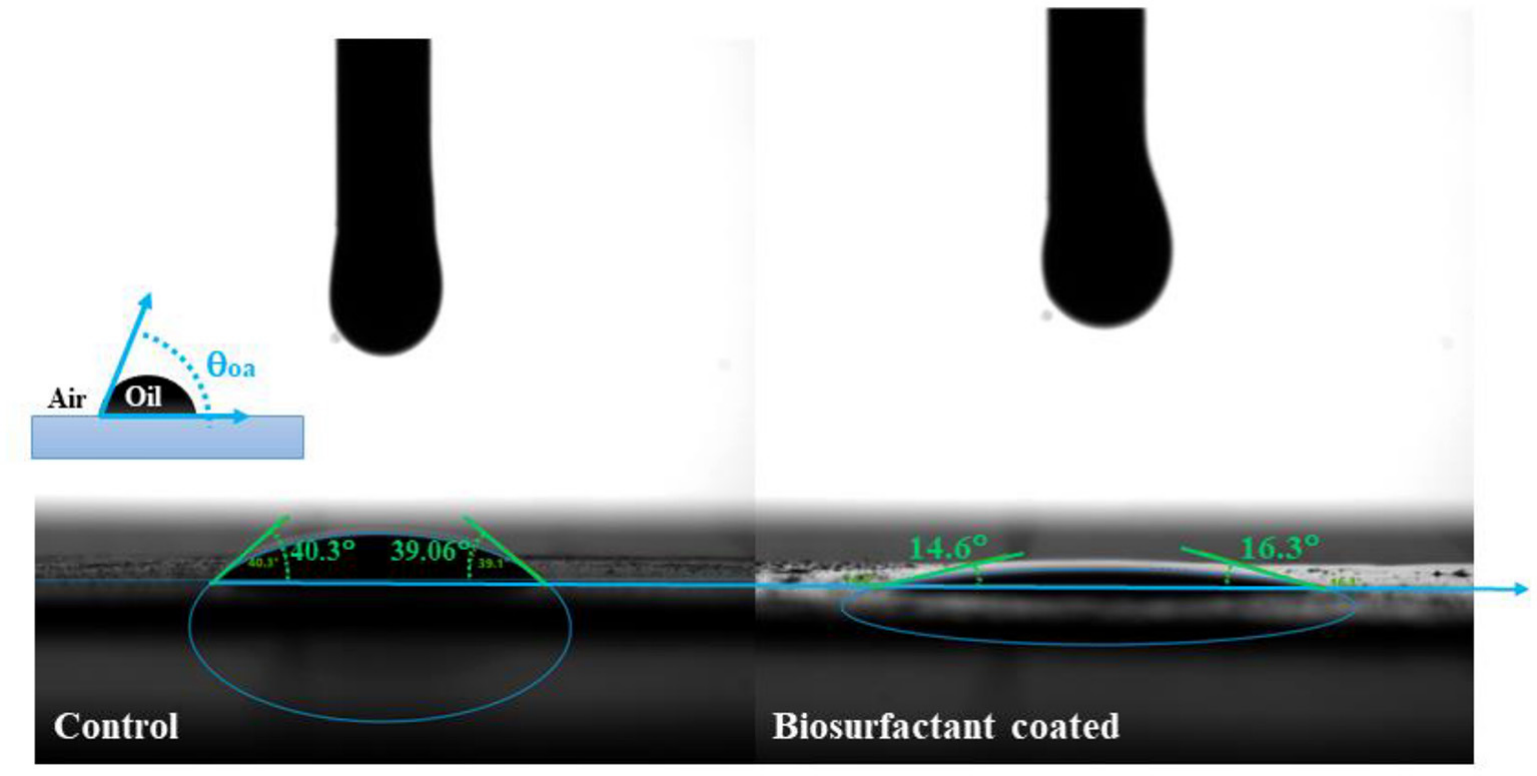
Oil–water contact angle measurement with and without a biosurfactant.

The surface tension of the produced biosurfactant was 30.5 mN/m. The interfacial tension of the produced biosurfactant was below the detection limit of the equipment, indicating that the oil–water interfacial tension of the biosurfactant at 1 CMC was below 1 mN/m. The oil–water contact angles with the existence of a bioherder were 14.6° and 16.3° for the left and right angles, respectively, much lower than that of the control (i.e., 40.3° and 39.06° for the left and right angles, respectively) (Figure 2). Recently, the mechanism of oil-herding agents has been identified (Huang et al., 2019). Once the oil is released into the marine environment, the high water–air interfacial tension (72 mN/m) causes oil slicks, whose water–oil and air–oil interfacial tension is 25 mN/m (Venkataraman et al., 2013), to spread quickly and form a thin layer on the water surface to reach equilibrium at the interface. The addition of herding agents at the oil slick edges could quickly reduce the oil–water interfacial tension (even lower than 1 mN/m) around the oil slick edges, making this value much lower than the values in the center region. Therefore, a negative spreading coefficient was generated, which facilitates the thickening of the oil slicks to reach a new equilibrium. Therefore, the low surface tension and oil–water interfacial tension of the biosurfactant generated in this study make it a good herder candidate for oil spill treatment.

### Bioherder Performance Evaluation

**Figure 3:**
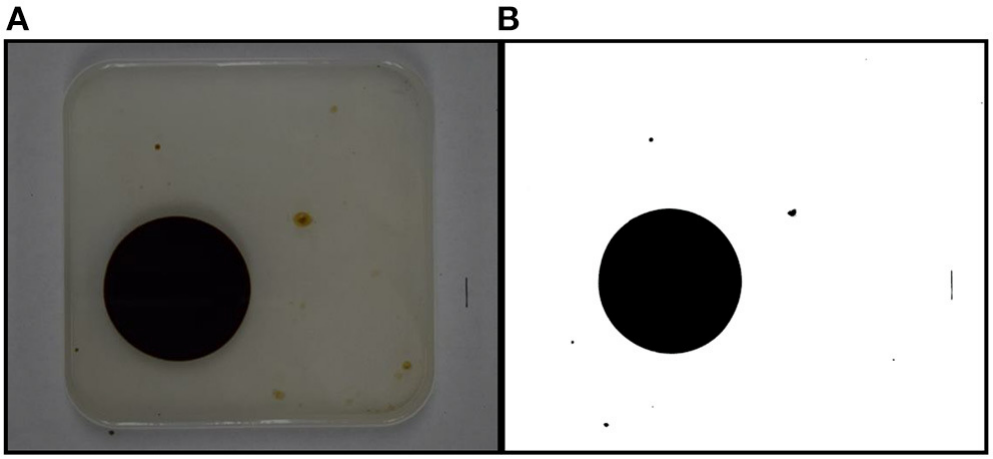
An example of calculating the oil slicks using Image J (*t* = 20 min). **(A)** the real picture took during herding experiment; **(B)** The picture processed by Image J.

Bioherder performance was evaluated based on herding effectiveness and efficiency. Herding effectiveness is the area change rate between time 0 and 20 min. Herding efficiency is the area change rate in 1 min after introducing a bioherder into the system. Herding effectiveness was calculated using Equation (1). Area values were measured from the photos captured by the overhead camera. Each photo was converted to black and white images, and pixels were measured using the software Image J. Every measurement is based on the same scale setting (10 mm) marked on the side of the testing pan. The initial area is the measurement of the picture shot right before the addition of a bioherder. The original and black and white images in Figure 3 show an example of the imaging process by Image J. The measurement of this oil slick for run # 8 is 4,470.23 mm^2^. The area of the oil slick at time *t* is measured and calculated following the same procedures.

**Figure 4:**
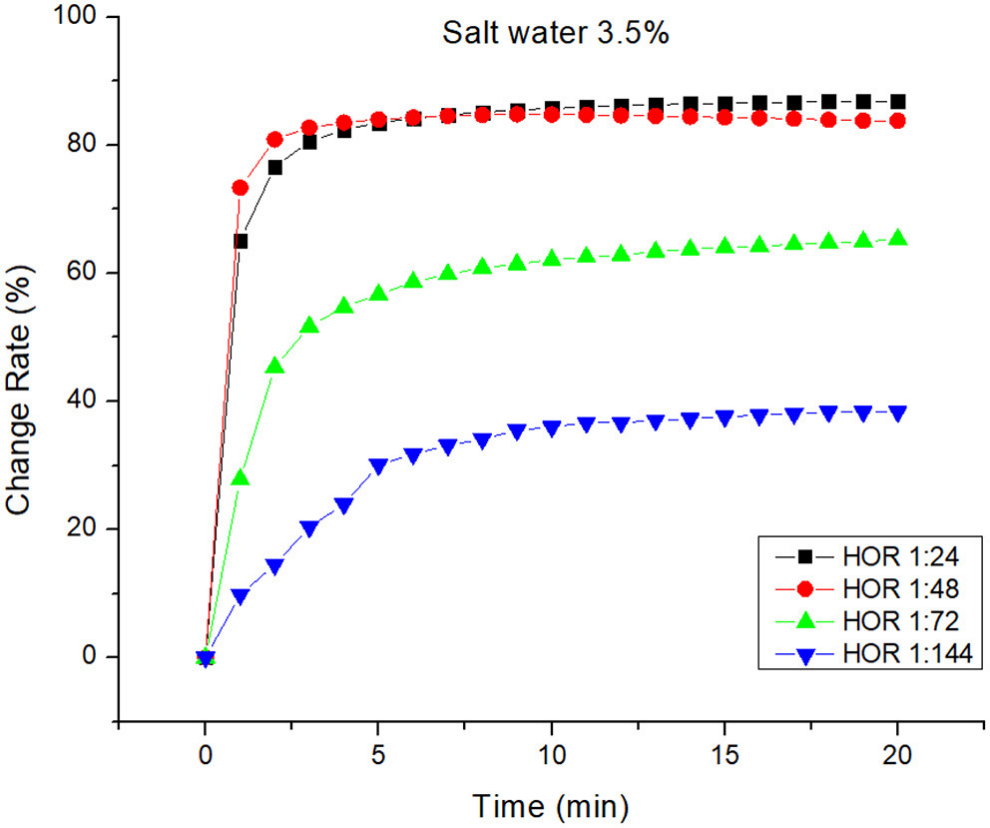
Herding efficacy of a bioherd in seawater at room temperature.

**Figure 5:**
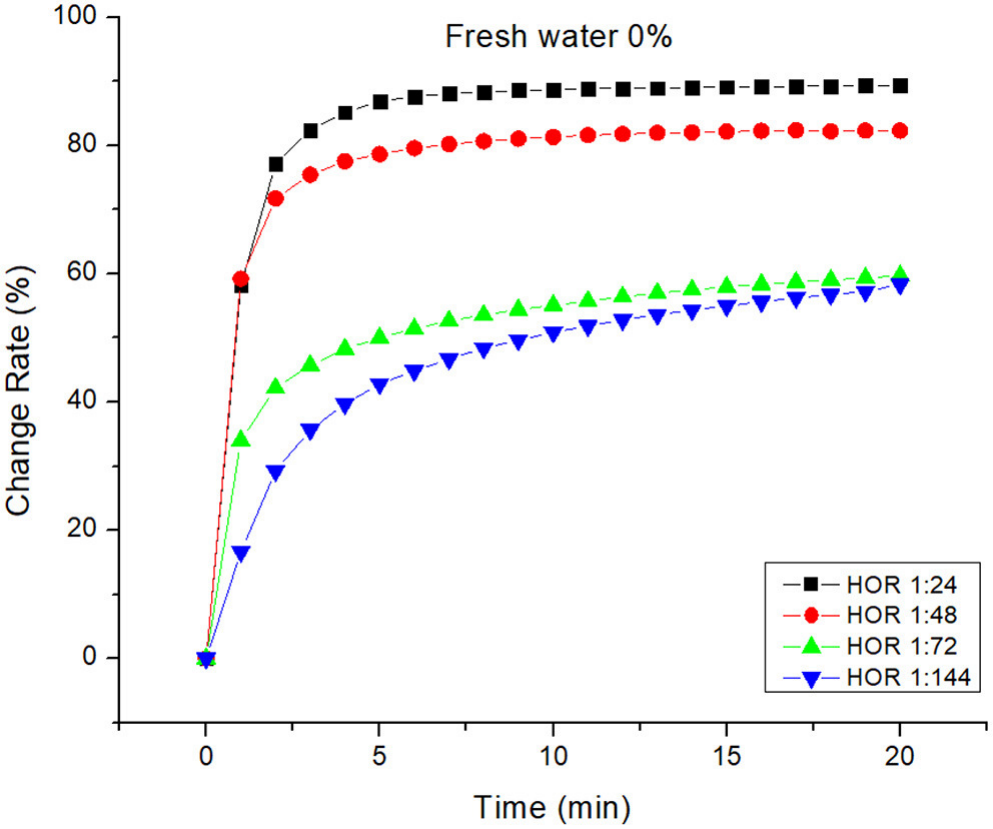
Herding efficacy of a bioherd in fresh water at room temperature.

The response of this design is the herding effectiveness of the generated bioherder. The change in herding effectiveness for differences in HOR in artificial seawater and freshwater are illustrated in Figures 4, 5, respectively. For differences in water salinity at the same temperature, both the figures indicate the same trend that herding effectively goes up with increasing HOR. Herding effectiveness for both freshwater and saltwater increased sharply in the first 3 min and then gradually increased.

As can be seen in Figure 4, the difference in the area change rate with large HOR is smaller than that with low HOR. At 20 min, the oil slick area change rate was declined by 3.5% (from 86.82% to 83.82%) when the HOR was decreased by half (from 1:24 to 1:48). The oil slick change rate was lowered by 25% when the HOR was decreased to 1/3 of its original level (from 1:24 to 1:72). Simultaneously, the change rate was dropped by 56% when the HOR was decreased to 1/4 of its original level (from 1:24 to 1:144). On the other hand, the impact of HOR on freshwater herding effectiveness was slightly different (Figure 5). At 20 min, the oil slick area change rate was declined by 7% (from 89.33% to 82.32%) when the HOR was decreased from 1:24 to 1:72. It seems that there was no significant change in herding effectiveness when the HOR was dropped by 1/3 or 1/4. This indicates that the herder performance itself has a greater impact on herding effectiveness. When a higher performance herder is used, only a small amount is needed.

**Figure 6:**
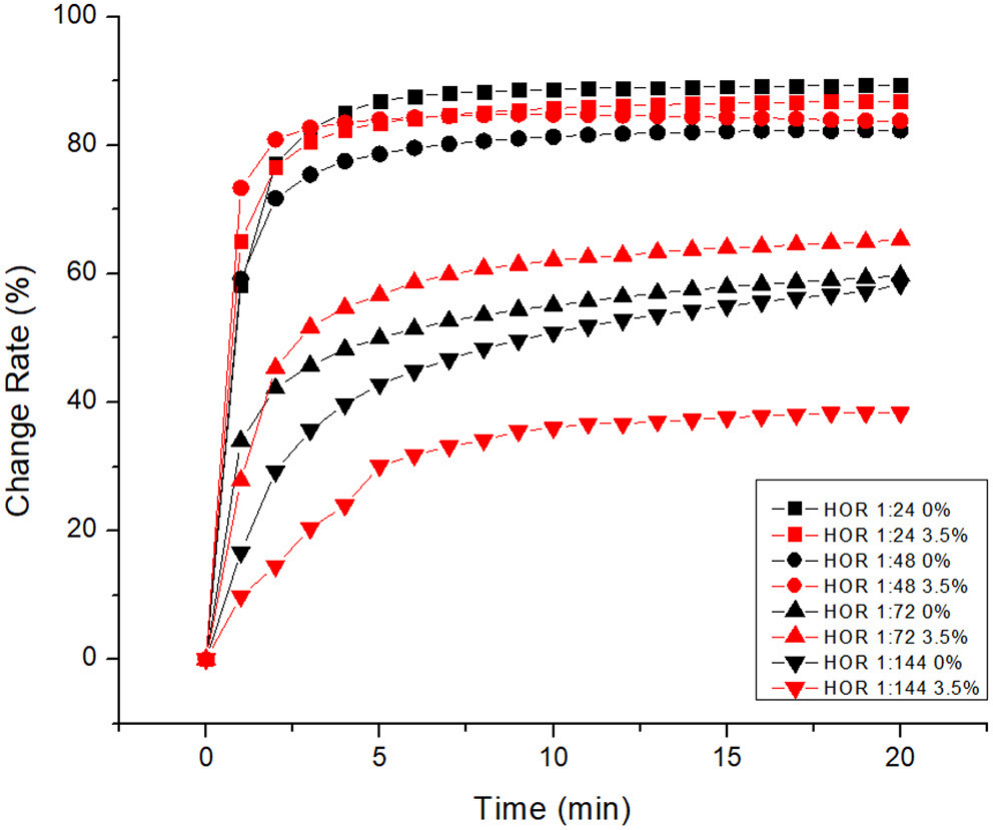
Impact of salinity on bioherding effectiveness at room temperature.

Figure 6 shows the impact of salinity on bioherding effectiveness for a different level of HOR. The most effective herding rate for 20 min is 89.33% at room temperature for freshwater. The most rapid bioherder reaction in the first minute is 65% in saltwater at room temperature. It seems that the herding increases with decreasing HOR. There is no significant difference in the change rate when changing the HOR from 1:24 to 1:72 in both freshwater and saltwater. The impact of a herder itself is higher than the impact of salinity. There is a significant change of salinity on the change rate when the HOR is low. Salinity has a significant impact on performance when a smaller herder dose is applied to the system. This indicates that, when herding agents are used in saltwater, the number of herds per unit of oil should be increased.

The performance of bioherders and chemical herders was compared using the same laboratory setting and procedures. Hydrocarbon-based chemical herder span 20, silicon-based chemical herder silsurf, and crude bioherder produced by *Rhodococcus* were used at the same dose following standard protocols. According to Buist et al. (2010), the dose of herder applied to the water surface is 150 μg/m^2^. This dose converted this customized testing system to 2.16 μl, making the HOR 1:333 (Table 3). This HOR was not set for bioherder performance in the last session because it required a large-scale pan to allow for oil spreading. This series of experiments was carried out under steady room conditions with no wind or heat interpretation. The performance evaluation period was shortened because the chemical herder reacted rapidly. The chemical herder rapidly increased the oil slick thickness and tended to push the oil toward the wall of testing pans. It can be assumed that a chemical herder reacts quickly and interacts with the oil slick before it forms a monolayer on the water surface. This needs to be proven by testing in a large-scale system. As can be seen in Table 3, the change rate of the silsurf herder is higher than that of the span and a bioherder.

**Table 3:** Performance comparison of chemical and bioherders under the same conditions.

	**Span**	**Silsurf**	**Bioherder**
Temperature °C	24	24	24
Herder dose μl	2.16	2.16	2.16
HOR	1:333	1:333	1:333
Water salinity %	3.5	3.5	3.5
Change rate %	89	93	74

### Bioherder Applications for Marine Oil Spill Response

The impacts of environmental factors on herding performance and their interactions are evaluated using the DOE results. The five factors of the experimental design and results are summarized in Table 4. Two levels of factor design analysis were carried out for 1 (efficiency) and 20 min (effectiveness). The analysis of variance (ANOVA) analysis was performed with a confidence level of 0.05. According to Table 5, the significant effects are A (temperature), D (herder dose), and their interaction AD (*p* < 0.0001), whereas factors C (oil amount), E (adding location), the other two factor interactions, and other quadric terms are not significant. The lack of fit is not significant, indicating that the model is a good fit. In addition, the difference between the adjusted *R*^2^ (0.8790) and predicted *R*^2^ (0.8059) is <0.2. The Adeq precision value of thie generated model is 16.0870, which indicates an adequate signal. A signal-to-noise ratio greater than 4 is desirable. According to Table 6, the significant effects are A (temperature) and D (herder dose) (*p* < 0.0001). The main factors B (salinity), C (the amount of oil), E (adding location), the other two factor interactions, and other quadric terms are not significant. The lack of fit is not significant, indicating that the model is a good fit. In addition, the difference between the adjusted *R*^2^ (0.8633) and the predicted *R*^2^ (0.7344) is <0.2. The precision of Adeq is 14.0966, which indicates an adequate signal. A signal-to-noise ratio >4 is desirable. This model can be used to navigate the design space.

**Table 4:** Design of experiments (DOE) experimental variables and results.

		**Levels of variables**					**Response**	
**Standard order**	**Actual run**	**Temperature (**°**C)**	**Salinity (%)**	**Oil (μl)**	**Herder (μl)**	**Approach**	**Change rate (%)**	
							**1 min**	**20 min**
19	1	14	1.75	540	10	1	24.31	81.75
20	2	14	1.75	540	10	2	34.02	87.06
3	3	4	3.5	360	5	1	4.16	17.25
12	4	24	3.5	360	15	1	65.16	86.82
5	5	4	0	720	5	1	5.37	22.34
18	6	14	1.75	540	10	2	20.24	69.77
4	7	24	3.5	360	5	2	27.86	65.36
6	8	24	0	720	5	2	16.65	58.35
11	9	4	3.5	360	15	2	16.55	56.00
2	10	24	0	360	5	1	34.02	59.74
7	11	4	3.5	720	5	2	5.99	40.92
17	12	14	1.75	540	10	1	35.68	75.24
9	13	4	0	360	15	1	2.75	47.84
1	14	4	0	360	5	2	−0.21	44.97
8	15	24	3.5	720	5	1	9.79	38.38
14	16	24	0	720	15	1	59.22	82.32
10	17	24	0	360	15	2	58.17	89.33
13	18	4	0	720	15	2	0.32	51.45
16	19	24	3.5	720	15	2	73.38	83.82
15	20	4	3.5	720	15	1	35.78	66.23

**Table 5:** The analysis of variance (ANOVA) table for factorial design at 1 min.

**Source**	**Sum of squares**	**df**	**Mean square**	**F-value**	**p-value**	
Model	9015.30	5	1803.06	28.61	<0.0001	Significant
A-Temperature	4675.69	1	4675.69	74.18	<0.0001	
B-salinity	243.21	1	243.21	3.86	0.0697	
D-herder dose	2695.71	1	2695.71	42.77	<0.0001	
AD	1016.21	1	1016.21	16.12	0.0013	
BD	384.48	1	384.48	6.10	0.0270	
Residual	882.43	14	63.03			
Lack of fit	723.02	12	60.25	0.7559	0.6974	Not significant
Pure error	159.41	2	79.71			
Cor total	9897.73	19				
Adjusted R^2^	0.8790					
Predicted R^2^	0.8059					
Adeq precision	16.0870					

**Table 6:** The ANOVA table for factorial design at 20 min.

**Source**	**Sum of squares**	**df**	**Mean square**	**F-value**	***p*-value**	
Model	6461.82	4	1615.45	27.85	<0.0001	Significant
A-Temperature	2945.92	1	2945.92	50.79	<0.0001	
D-herder dose	2930.08	1	2930.08	50.51	<0.0001	
E-Location	300.00	1	300.00	5.17	0.0406	
DE	346.96	1	346.96	5.98	0.0295	
Curvature	1541.61	2	770.81	13.29	0.0007	
Residual	754.08	13	58.01			
Lack of fit	583.55	11	53.05	0.6222	0.7559	Not significant
Pure error	170.53	2	85.27			
Cor total	8757.51	19				
Adjusted R^2^	0.8633					
Predicted R^2^	0.7344					
Adeq precision	14.0966					

Herders tend to move from a higher concentration environment (i.e., the water environment) to a low concentration environment (i.e., the oil layer). The aforementioned concentration gradient also triggers the retraction of the spread oil and thickens the oil layer (Gupta et al., 2015). The acceleration of herding process and enhancement of herding efficiency with increased dosage of a herding agent were verified here. The contribution of temperature to the surface activities of biosurfactants has been explained in detail by Zhu et al. (2021). By affecting the mobility of biosurfactant fatty acid tails, their expansion on the liquid surface, and the micellar structures formed in this process, a reduced temperature could lead to increased air–water interfacial tension and a higher critical micellar concentration of biosurfactants (Cao et al., 2022). Therefore, it is reasonable to assume that herding efficiency could be hindered. In addition, the increased viscosity of the spilled oil could further reduce the efficiency of herding agents (Firooz and Chen, 2011).

### Feasibility of Biosurfactants as Bioherders

Herding agents have been considered as an effective oil spill treatment agent to improve oil spill response. Research efforts have been continuously centered on the development of herding agents with improved performance and reduced environmental footprint. As a biological counterpart to chemical surfactants, biosurfactant-based bioherders have received increasing attention. Before their application in the field, technical and economic feasibility analyses are desired.

As this study indicates, the reduction of air–water interfacial tension plays an important role in the herding process. Table 1 compares the surface activities and toxicities of trehalose lipids and chemical surfactants. Trehalose lipids could greatly reduce the air–water and oil–water interfacial tension, which is comparable to that of chemical surfactants. In the meantime, trehalose lipids tend to possess much lower ecological toxicity than chemical surfactants. It is worth mentioning that trehalose lipids possess even lower toxicity than other popular biosurfactants, such as rhamnolipid and surfactin. Therefore, the replacement of biosurfactants for chemical ones is technically feasible.

The high production cost of biosurfactants becomes the major hurdle faced by the environmental industry for the development of bioherders. The retail price for the biosurfactants that are available at the market ranges from $20 to 600/kg (Chong and Li, 2017; Zhu et al., 2020), much higher than that of chemical surfactants (i.e., $20 to 600/kg) (Chong and Li, 2017). However, breakthroughs in recent advances shed light on reducing the production cost reduction of biosurfactants. The use of low-cost substrates, such as waste and by-products, could reduce 10–30% of the production cost of biosurfactants (Kosaric and Sukan, 2014). For example, the reported production cost for sophorolipid is $2.95/kg (Dhanarajan and Sen, 2014), whereas the estimated cost for sophorolipid production could be reduced to around $0.1–0.22/L with sugarcane molasses, steep corn liquor, and soy waste as substrate (Freitas et al., 2016). Biosurfactant production costs could be further reduced through the development of engineering strains and innovative bioreactors. Therefore, the future application of biosurfactants as bioherders could be promising.

## Conclusion

This study has demonstrated that biosurfactants can be used as herding agents for marine oil spill response. Temperature and the herder/oil ratio were found to exert high impacts on herding performance. The biosurfactant produced by *R. erythropolis* M25 as a herding agent was examined, and the influence of environmental and operational factors (i.e., temperature, herder dose, amount of oil spilled, water salinity, and the site of operation) on its performance was investigated. A five-factor fractional design was applied to investigate the importance of these factors and their impact on herding effectiveness and efficiency. The results of this study showed that higher temperature and a larger amount of herder could result in a higher rate of oil slick thickness change. Differences in water salinity under the same temperature led to the same trend; that is, the herding process effectively goes up with increasing HOR. Further large-scale testing needs to be conducted to evaluate the applicability of the developed bioherder in the field.

## Data Availability Statement

The original contributions presented in the study are included in the article/supplementary material, further inquiries can be directed to the corresponding author/s.

## Author Contributions

MY and ZZ are mainly responsible for the research initiation, experimentation, result analysis, and generation of the manuscript. BC helps to conduct the data analysis and polish the manuscript. YC helps to edit the manuscript. BZ is the principal investigator of the manuscript involved research project and funds and is mainly responsible for supervising MY and ZZ throughout the research and finalizing the manuscript. All authors contributed to the article and approved the submitted version.

## Funding

This work was funded by Canada Research Chairs (CRC) Program (#950-231554), Canada Foundation for Innovation (#36677), Natural Sciences and Engineering Research Council of Canada (#RGPIN-2018-05378), and Fisheries and Oceans Canada (DFO), MPRI #1.02.

## Conflict of Interest

The authors declare that the research was conducted in the absence of any commercial or financial relationships that could be construed as a potential conflict of interest.

## Publisher's Note

All claims expressed in this article are solely those of the authors and do not necessarily represent those of their affiliated organizations, or those of the publisher, the editors and the reviewers. Any product that may be evaluated in this article, or claim that may be made by its manufacturer, is not guaranteed or endorsed by the publisher.
